# Study on Failure Pressure Prediction of Double Corrosion Defects in Oil and Gas Pipelines

**DOI:** 10.3390/ma19102166

**Published:** 2026-05-21

**Authors:** Lihua Chen, Guoxing Yu, Lele He, Youjia Zhang, Shuqin Zheng, Xu Wang, Yanru Wang, Lei Zhou

**Affiliations:** 1 School of Civil Engineering, Chongqing Vocational Institute of Engineering, Chongqing 402260, China; 2 School of Civil Engineering, Chongqing Jiaotong University, Chongqing 400074, China; cyhl0901@163.com (L.H.); xuwang@cqjtu.edu.cn (X.W.); lixuezl@tju.edu.cn (L.Z.); 3 Chief Engineer Office, Chongqing CISDI Engineering Consulting Co., Ltd., Chongqing 400013, China; guoxing.yu@cisdi.com.cn; 4 School of Civil Engineering and Architecture, Northeast Electric Power University, Jilin 132012, China; 5 Key Laboratory of Intelligent Lifeline Protection and Emergency Technology for Resident ATY, Wenzhou University of Technology, Wenzhou 325000, China; 6 School of Civil Engineering, Taizhou University, Jiaojiang 318000, China; yanrupiaoyang@163.com

**Keywords:** multi-point corrosion, defect interaction, critical spacing, failure pressure, oil and gas pipelines

## Abstract

Corrosion defects in actual oil and gas pipelines tend to occur in clusters, and the mechanical interaction between adjacent defects alters the local stress field, thereby affecting the overall failure behavior and load-bearing capacity of the pipeline. Existing pipeline integrity assessment codes often adopt simplified treatments (e.g., the defect projection method) for multi-point corrosion, which fail to fully consider the influence of defect spacing and may lead to inaccurate (overly conservative or unsafe) evaluation results. To reveal the interaction mechanism of double-point corrosion, this paper takes X65 pipeline as the research object, establishes finite element models for two typical types of double-point uniform corrosion defects (planar axial and circumferential arrangements), and systematically investigates the effects of defect spacing, depth, and length on the failure pressure. The results show that the axial spacing has a significant influence on the failure pressure, and a critical spacing (60 mm) exists. When the spacing is smaller than the critical value, the failure pressure decreases by up to 18% compared to the single-defect case, and the critical spacing decreases with increasing corrosion depth. In contrast, the influence of circumferential spacing is much weaker and can be neglected. Based on extensive numerical results, empirical fitting formulas for the failure pressure of axially and circumferentially spaced double-point corrosion are provided. The goodness-of-fit (R^2^) reaches 0.97805 and 0.99964, respectively. This study clarifies the mechanical interaction mechanism of double-point corrosion and can serve as a reference for the residual strength assessment of pipelines containing adjacent corrosion defects.

## 1. Introduction

As the arteries of energy transportation, oil and gas pipelines under safe and stable operation are directly tied to the lifeline of national economy and public social security. The total mileage of oil and gas pipelines in China has exceeded 150,000 km, among which those constructed in the early stage have gradually entered the aging period. Pipeline failure accidents caused by corrosion, third-party damage, material deterioration and other factors occur frequently, resulting in heavy casualties and economic losses [1,2,3,4,5]. Statistical data on pipeline failures at home and abroad indicates that corrosion is one of the primary causes leading to pipeline malfunctions, accounting for 24.1% and 20.0% of pipeline accidents in China and the United States respectively. In recent years, a series of major accidents, such as the November 22 oil pipeline explosion in Qingdao in 2013 and the gas pipeline explosion in Kaohsiung, Taiwan China in 2014, were all directly caused by corrosion-induced thinning and rupture of pipelines. These incidents have highlighted the urgency of conducting residual strength assessment and failure prediction for corroded pipelines [6,7,8,9].

Numerical simulation has emerged as a pivotal approach in the research on the residual strength of corroded pipelines. Since Wang [10] first applied the finite element method to investigate the failure of corroded pipelines, relevant theories such as the plastic limit criterion [11,12] have been continuously advanced. Research on single-point corrosion defects has achieved in-depth progress, with scholars extensively exploring the influence of factors including corrosion dimensions (depth, length, width) and pipeline steel grades on failure pressure. However, multiple corrosion defects are commonly found in actual pipelines, and the interaction between adjacent defects can drastically alter the stress distribution and failure mode of pipelines, rendering the assessment of residual strength far more complex [13,14,15,16,17,18]. In response to this issue, scholars at home and abroad have begun to focus on the interaction effect of multi-point corrosion. For instance, Chen [19] conducted research on double corrosion defects in X80 pipelines and concluded that the increase in corrosion depth would intensify the interaction between defects when the axial spacing is fixed. Yang Yanhua et al. [20] pointed out that the depth of axially distributed double pitting corrosion exerts the most significant impact on failure pressure. Chen et al. [21] once proposed an assessment procedure for interactive defects in X80 pipelines, yet its universality is limited. Chauhan [22] verified through full-scale tests that existing assessment methods may be overly conservative for pipelines with interactive defects. Overall, existing research still lacks systematic analysis on the impacts of parameters such as the relative axial and circumferential positions and dimension combinations of defects on the internal pressure bearing capacity in the case of double-point corrosion, especially under the uniform corrosion morphology. Additionally, there is a shortage of failure pressure prediction models with high accuracy and wide applicability.

Against this backdrop, this paper takes X65 grade pipelines with double uniformly corroded defects under internal pressure loading as the research subject and develops a nonlinear numerical model by means of the ABAQUS 2024 finite element software. The research aims to systematically explore the interaction mechanism between double-point corrosion defects, analyze the influence laws of key parameters such as corrosion depth, length, width and defect spacing on the failure pressure of pipelines, and further establish a more accurate predictive expression for the failure pressure of pipelines with double uniformly corroded defects. It is expected to provide theoretical foundations and references for the integrity assessment and safety maintenance of such pipelines.

## 2. Finite Element Model and Research Method for Defect Interaction

### 2.1. Establishment of the Double-Point Defect Model

#### 2.1.1. Finite Element Analysis Details Under Internal Pressure Loading

Based on the geometric model of double-point uniform corrosion defects, a three-dimensional nonlinear finite element model was established to analyze the failure behavior of pipelines under internal pressure loading. Using the ABAQUS 2024 software, monotonically increasing internal pressure was applied to both axially and circumferentially spaced double-point corrosion defects, and the influence of defect spacing, depth, length and other parameters on the failure pressure was investigated.

(1) Mesh Configuration

The model was constructed using hexahedral solid elements (C3D8). A coarse mesh size of 25 mm × 25 mm was used in regions far from the corrosion defect, while the defect region and its vicinity were refined to 15 mm × 15 mm, with three layers of elements through the wall thickness.

(2) Mesh Sensitivity Analysis

A mesh sensitivity analysis was conducted using a typical X65 pipeline scenario (relative depth 0.2, corrosion length 200 mm, axial spacing 40 mm). Three mesh schemes were compared: coarse mesh (20 mm × 20 mm, 2 layers through thickness), medium mesh (15 mm × 15 mm, 3 layers), and fine mesh (10 mm × 10 mm, 4 layers). The results showed that the relative error in failure pressure between the medium and fine meshes was 1.2%, while the error between the coarse and fine meshes was 4.5%. Considering both accuracy and computational efficiency, the medium mesh scheme was adopted for all subsequent calculations.

(3) Analysis Settings

Boundary conditions: An axial displacement constraint was applied at one end of the pipe, while the other degrees of freedom were left free.

Loading: A monotonically increasing uniform pressure was applied to the inner wall. Additional loads such as self-weight and soil pressure were neglected.

Failure criterion: The plastic failure criterion was adopted. The pipe was considered to have burst when the maximum von Mises equivalent stress reached the tensile strength of the material, and the corresponding internal pressure was taken as the failure pressure.

Nonlinearity handling: Geometric nonlinearity (NLGEOM) was enabled, and the Newton–Raphson iterative method was used for solving.

The above settings ensure the reliability and accuracy of the finite element calculations for double-point corrosion defect pipelines under internal pressure.

#### 2.1.2. Geometric Description of Double-Point Corrosion Defects

**Figure 1:**
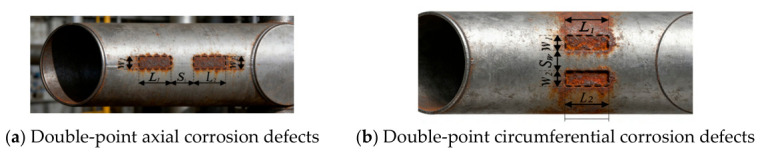
Schematic diagram of double-point uniform corrosion defects.

In practical engineering, pipeline corrosion is rarely an isolated occurrence, and there exists an interaction among the geometric parameters of corrosion defects. At present, research on the interaction and evaluation of two or more corrosion defects on buried pipelines remains relatively inadequate. Double-point uniform corrosion defects refer to two adjacent corrosion areas with similar characteristics on the pipe surface, which usually present as defects with relatively uniform shape, depth and distribution, exerting a crucial impact on the structural integrity and failure pressure of pipelines. According to the relative positions of the two defects, double-point uniform corrosion defects can be mainly classified into axially distributed double-point corrosion defects and circumferentially distributed double-point uniform corrosion defects, with the schematic diagram shown in Figure 1. In the figure, $L_{1}$ and $L_{2}$ denote the corrosion lengths (mm), $W_{1}$ and $W_{2}$ represent the corrosion widths (°), $S_{l}$ stands for the axial spacing(mm), and $S_{w}$ indicates the circumferential spacing (°).

It should be noted that the corrosion defect is simplified as a rectangular profile (Figure 1), assuming a zero filet radius (sharp corner) at the defect bottom. This simplification aligns with current engineering codes and is intended to yield conservative predictions. Actual corrosion pits have a transitional filet at the bottom, and its effect should be examined via sensitivity analysis in future work.

### 2.2. Interaction of Axially Distributed Double-Point Corrosion Defects

#### 2.2.1. Corrosion Depth and Axial Spacing

**Figure 2:**
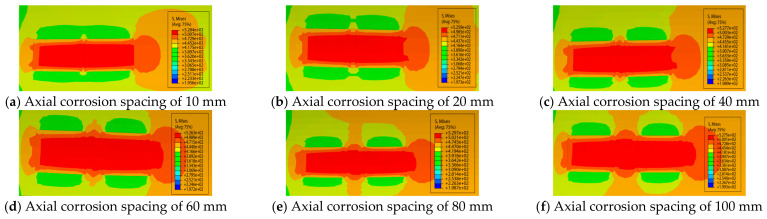
Stress nephograms of corroded pipelines with different spacings at M=0.2.

Assume that there exist two corrosion defects with identical geometric dimensions along the pipeline axial direction, taking X65 grade steel pipelines as the research case. The defect depth is expressed by the relative depth $M\left(M=d/t\right)$, where d and t are the depth of the corrosion defect and the wall thickness of the pipe, respectively. The corrosion lengths $L_{1}$ and $L_{2}$ are 200 mm, and the corrosion width is fixed at 20°. The axial spacing between the two defects is denoted by the parameter $S_{l}$ with the specific values listed in Table 1. Figure 2 presents the stress nephograms corresponding to different axial spacing values when the relative depth of the corrosion defects is 0.2.

**Figure 3:**
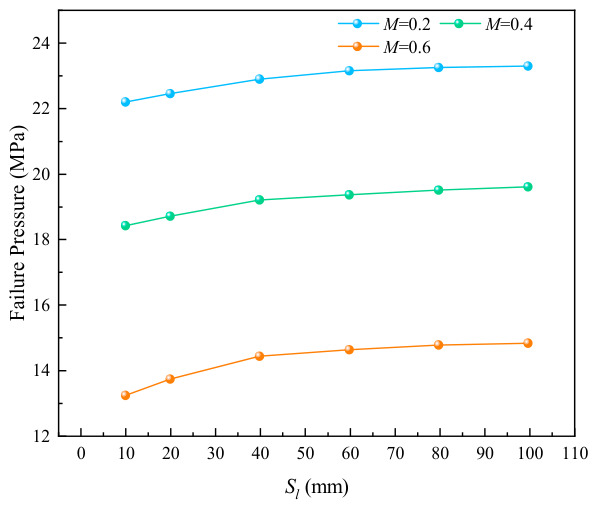
Variation in failure pressure with $S_{l}$ at different corrosion depths.

It can be seen from Figure 2 that when the axial spacing between double-point corrosion defects is small, the maximum stress is mainly distributed in the corrosion defect areas and the region between them. Owing to the mutual interaction between the defects, the pipeline failure is highly likely to occur at the position between the two defects when the spacing increases to a certain critical value. The curves of pipeline failure pressure versus corrosion axial spacing at different relative corrosion depths, calculated using the SY/T 6151 method [23], are shown in Figure 3.

Based on the finite element simulation cases listed in Table 1 (X65 pipeline, corrosion length 200 mm, corrosion width 20°, and axial spacing ranging from 10 to 100 mm), the failure pressures of pipelines with double-point uniform corrosion defects were calculated. For comparison, the failure pressures of pipelines with single-point corrosion defects at the corresponding corrosion depths $\left(M=0.2,\;0.4,\;0.6\right)$ were also computed using the same finite element model (specific values are given in Table 2). The results show that at each corrosion depth, the failure pressure of the double-point corroded pipeline is lower than that of the single-point corroded pipeline. Taking an axial spacing of 10 mm as an example, when M=0.2, 0.4, 0.6, the failure pressures of the double-point corroded pipeline are reduced by 1.9 MPa, 3.0 MPa and 3.9 MPa, respectively, compared with those of the single-point corroded pipeline (data derived from the simulation results in Table 1 and Table 2). Furthermore, when the spacing between the two corrosion defects reaches a certain critical value, the failure pressure tends to stabilize. To further judge whether the interaction between axially distributed double-point corrosion defects occurs and thus affects the pipeline failure pressure, this study adopts $\lambda =\left|P_{i+1}-P_{i}\right|/P_{f}=0.01$ as the criterion for the critical state, where the interaction between adjacent defects can be neglected. At a relative corrosion depth of 0.2, $\lambda =\begin{array}{r} 0.0041 \end{array}$, corresponding to a critical axial spacing of 60 mm for double-point corrosion defects; at a relative corrosion depth of 0.4, $\lambda =\begin{array}{r} 0.007 \end{array}$, with the critical spacing being 40 mm. This indicates that the critical spacing of double-point corrosion defects decreases gradually with the increase in relative corrosion depth.

#### 2.2.2. Corrosion Length and Axial Spacing

**Figure 4:**
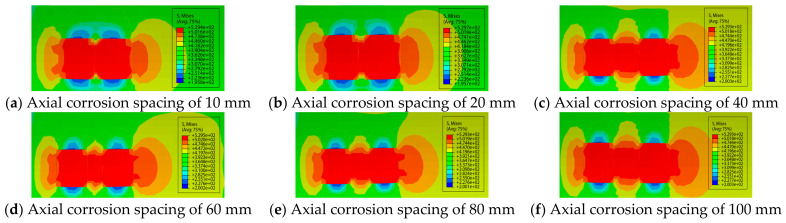
Stress nephograms of corroded pipelines with different spacings at N=1.0

Consistent with the specifications in the previous subsection, the relative corrosion length $N(N=L/\sqrt{Dt})$ and the relative axial spacing $S_{l}$ were selected as the independent variables, while the relative corrosion depth M and corrosion width were set as constant parameters. The specific corrosion parameters are presented in Table 3. Figure 4 shows the stress nephograms at different axial spacing values when the relative depth of corrosion defects is 0.2.

**Figure 5:**
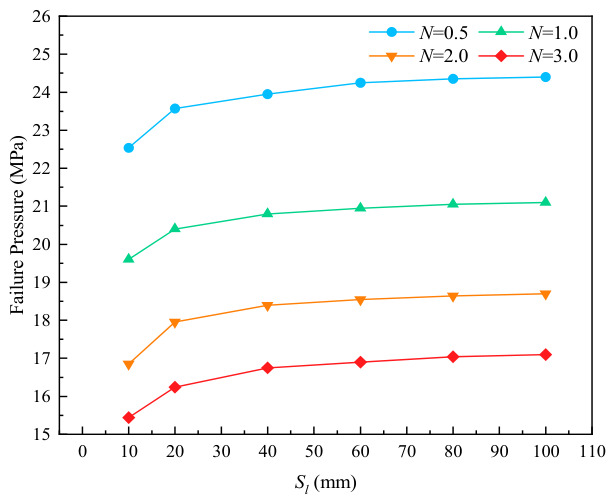
Variation in failure pressure with $S_{l}$ at different corrosion lengths.

Based on the geometric parameters listed in Table 2, numerical calculations were performed using the ABAQUS finite element model for axially adjacent double-point corrosion defects, and the interaction relationship between the defects was fitted, as shown in Figure 5. It can be seen from the figure that the variation in corrosion length with axial corrosion spacing is similar to that of corrosion depth. The failure pressure of the pipeline increases with the rise in the relative axial spacing between two axially adjacent corrosion defects, and gradually tends to a constant value in the later stage. For example, at the relative corrosion lengths of 0.5, 1.0, 2.0 and 3.0, the corresponding critical spacings are 60, 40, 40 and 40 mm respectively, and the calculated critical spacing λ values are 0.003, 0.006, 0.007 and 0.008 in sequence. With the increase in corrosion length, the critical interactive spacing of double-point corrosion decreases gradually [24].

### 2.3. Interaction of Circumferentially Distributed Double-Point Corrosion Defects

#### 2.3.1. Corrosion Depth and Circumferential Spacing

**Figure 6:**
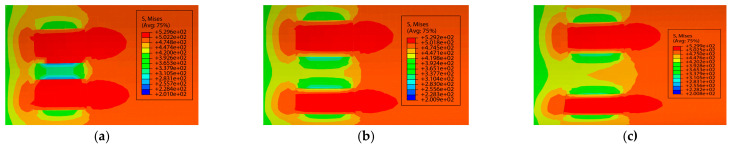
Stress nephograms of corroded pipes with different spacings at M=0.2. (**a**) Relative circumferential corrosion spacing of 10°, (**b**) Relative circumferential corrosion spacing of 20°, (**c**) Relative circumferential corrosion spacing of 30°.

The pipeline parameters were kept consistent with those for axially distributed double-point corrosion defects. The distance between circumferentially distributed corrosion defects was defined by the rotational angle $S_{w}$ around the pipeline central axis, with the specific corrosion parameters listed in Table 4. Figure 6 presents the stress nephograms under different circumferential spacing conditions when the relative depth of the corrosion defects was 0.2.

**Figure 7:**
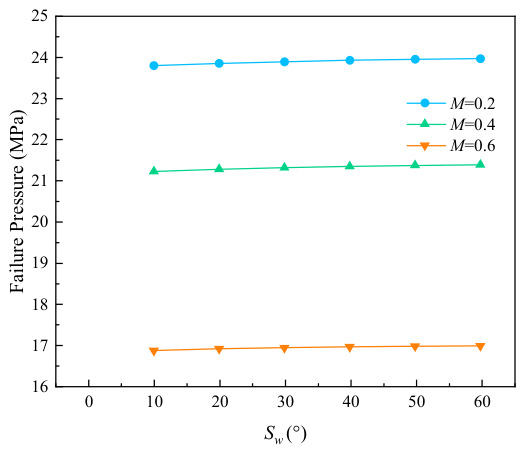
Variation in failure pressure with $S_{w}$ under different corrosion depths.

**Figure 8:**
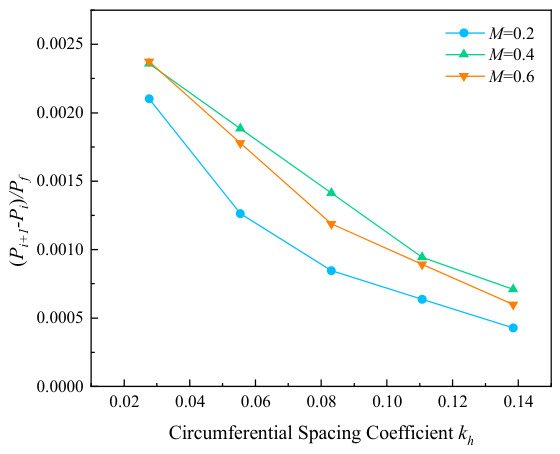
Variation in failure pressure with $k_{h}$ under different corrosion depths.

The variation curves of failure stress with circumferential corrosion spacing for corroded pipes with different relative corrosion defect depths are presented in Figure 7. It can be seen from the figure that circumferential double-point corrosion exerts a smaller influence on the failure pressure of pipes compared with axial double-point corrosion; the failure pressure varies in the range of 0.05 to 0.01 MPa with every 10 mm increase in corrosion spacing. To further explore the critical effect of the spacing between circumferential double-point corrosion defects with higher accuracy, the corrosion spacing was non-dimensionalized in this study as shown in Equation (1), and the ratio of $\left|P_{i+1}-P_{i}\right|/P_{f}$ was adopted for characterization. The variation in non-dimensional failure pressure with the circumferential spacing coefficient under different corrosion depths is illustrated in Figure 8. As indicated in the figure, the failure pressure changes significantly at a corrosion spacing of 10 mm for all corrosion depths, which may be attributed to the combined effect of double-point corrosion. With the increase in corrosion spacing, however, the failure pressure of the pipes tends to approach that of pipes with a single-point corrosion defect.

(1) $$k_{h}=S_{w}/360$$

where $k_{h}$—Circumferential spacing coefficient.

#### 2.3.2. Corrosion Length and Circumferential Spacing

**Figure 9:**
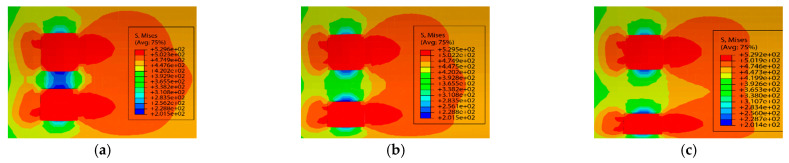
Stress nephograms of corroded pipes with different relative circumferential corrosion spacings at N=0.5. (**a**) Relative circumferential corrosion spacing of 10°, (**b**) Relative circumferential corrosion spacing of 20°, (**c**) Relative circumferential corrosion spacing of 30°.

Consistent with the defect parameters of axial double-point corrosion, the relative corrosion length $N(N=L/\sqrt{Dt})$ and relative circumferential spacing $S_{w}$ were selected as the independent variables, while the relative corrosion depth M and corrosion width were set as constants. The specific corrosion-related parameters are listed in Table 5. Figure 9 shows the stress nephograms under different circumferential spacing conditions with the relative length of corrosion defects fixed at 1.0.

**Figure 10:**
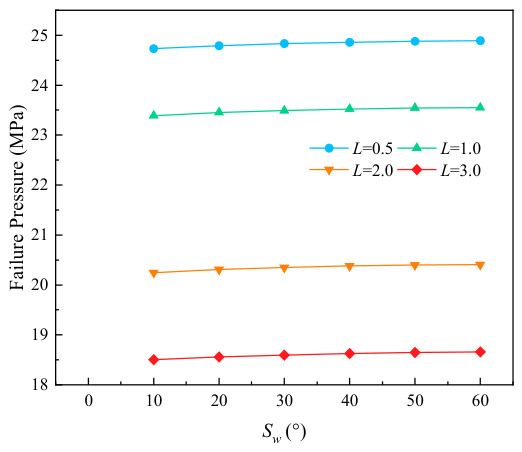
Variation in failure pressure with $S_{w}$ under different corrosion lengths.

**Figure 11:**
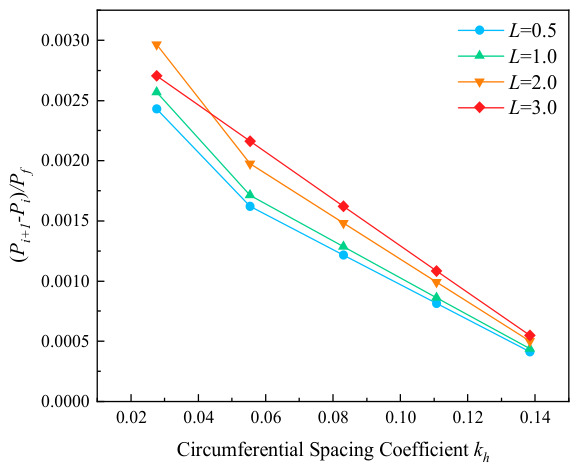
Variation in failure pressure with $k_{h}$ under different corrosion lengths.

The variation curves of pipe failure stress with relative circumferential corrosion spacing under different relative corrosion lengths are depicted in Figure 10. The results demonstrate that the change in circumferential spacing exerts a slight influence on the failure pressure under all corrosion length conditions, with the pressure variation range being approximately 0.05 to 0.01 MPa. Figure 11 further illustrates the relationship between non-dimensional failure pressure and circumferential spacing coefficient under various corrosion lengths. It can be observed that the non-dimensional failure pressure exhibits the most remarkable variation when the corrosion length coefficient is 3.0. In addition, all curves gradually tend to approach zero with the increase in circumferential corrosion spacing, which indicates that the interaction between two circumferential corrosion defects attenuates rapidly and becomes negligible as the spacing increases. Under such circumstances, the double-point corrosion can be approximately analyzed as two independent single-point corrosion defects.

## 3. Failure Behavior of Pipes with Double-Point Corrosion Defects

### 3.1. Calculation Method for Failure Pressure of Pipes with Axial Double-Point Corrosion

In practical engineering, the mutual interaction and restriction between corrosion defects are common phenomena. Based on the calculation formula for the failure pressure of pipes with single-point corrosion, this study therefore fitted the failure pressure of X65 grade pipes with axial double-point corrosion, and the fitting formula is given in Equation (2):

(2) $$P_{z}^{\prime }=aP_{f}+b\cdot {\left(M\right)}^{c}\cdot {\left(N\right)}^{d}\cdot {\left(Sl\right)}^{e}$$

where:

$P_{z}^{\prime }$—Failure pressure of axially double-point corroded pipeline (MPa);
$P_{f}$—Failure pressure of pipes with single-point corrosion (MPa);
a, b, c, d, e—Fitting coefficients.

Through systematic comparative analysis of fitting accuracy across various parameter combinations, the optimal values of the fitting coefficients were determined, as detailed in Table 6. Based on the above analysis, a predictive model for the failure pressure of pipelines with axially aligned double corrosion defects was established, with its mathematical expression given in Equation [25]. Statistical evaluation indicates that the model achieves a coefficient of determination ($R^{2}$) of 0.97805, demonstrating strong agreement with the numerical simulation results. Consequently, the model can effectively characterize the failure pressure behavior of X65 pipeline steel under axially aligned double corrosion defect conditions.

(3) $$P_{z}^{\prime }=0.66975\cdot P_{f}+1.85298\cdot {\left(M\right)}^{-0.63971}\cdot {\left(N\right)}^{-0.33592}\cdot {\left(Sl\right)}^{0.12546}$$

### 3.2. Calculation Method for Failure Pressure of Pipes with Circumferential Double-Point Corrosion

Based on the calculation formula for the failure pressure of single-point corroded pipes, this study fitted the failure pressure of X65 grade pipes with circumferential double-point corrosion, and the corresponding fitting formula is presented in Equation (4):

(4) $$P_{h}^{\prime }=aP_{f}+b\cdot {\left(M\right)}^{c}\cdot {\left(N\right)}^{d}\cdot {\left(S_{w}\right)}^{e}$$

where:

$P_{h}^{\prime }$—Failure pressure of pipes with circumferential double-point corrosion (MPa);
a, b, c, d, e—Fitting coefficients.

Through systematic comparison of fitting accuracy across various parameter combinations, the optimal values of the fitting coefficients were determined, with the specific results presented in Table 7. Based on this determination, a predictive model was established for the failure pressure of pipelines containing circumferentially aligned double corrosion defects, with its mathematical expression provided in the corresponding equation. Statistical evaluation indicates that the model achieves an exceptionally high coefficient of determination ($R^{2}$) of 0.99964, reflecting near-perfect agreement with the numerical simulation data. Consequently, the model can accurately characterize both the ultimate load-bearing capacity and the failure pressure response of X65 pipeline steel under circumferentially aligned double defect conditions.

(5) $$P_{h}^{\prime }=0.984P_{f}+0.01558M^{-0.2054}\cdot N^{0.36155}\cdot k_{h}^{0.55876}$$

### 3.3. Comparison with Existing Studies on Double Corrosion Defects

To systematically compare the interaction behavior of double corrosion defects in pipelines of different steel grades, Table 8 summarizes the key corrosion parameters and failure pressure data reported by Yang Yanhua et al. (X100), Chen Fei (X80), and the present study (X65).

As shown in Table 8, all three studies confirm that corrosion depth is the primary sensitive parameter influencing failure pressure. Specifically, Yang Yanhua et al. established a quantitative relationship for X100 high-grade pipelines, wherein the failure pressure decreases by 1–2.5 MPa for every 2 mm increase in corrosion depth. For X80 pipelines, Chen Fei reported that the failure pressure varies from 16.4 to 25.1 MPa as the relative depth increases from 0.2 to 0.8. In the present study on X65 pipelines with a fixed corrosion width of 20°, the double-point failure pressures (at an axial spacing of 10 mm) corresponding to relative depths of 0.2, 0.4, and 0.6 are 18.1, 15.0, and 12.1 MPa, respectively. Comparison reveals that high-grade pipelines exhibit slightly higher absolute sensitivity to corrosion depth than medium- and low-grade pipelines, although the overall failure trends remain consistent. In addition, the three studies differ in the adopted corrosion width values: Yang Yanhua et al. and Chen Fei employed relatively small widths (2.6–7.9°), whereas the present study selected a larger width of 20° to examine the stability of the width effect. The results indicate that the influence of width on failure pressure is relatively limited. In summary, Table 8 provides a parametric comparison basis for the residual strength assessment of pipelines of different steel grades containing double corrosion defects.

## 4. Conclusions

This chapter presents a numerical simulation analysis of planar axial and circumferential double uniform corrosion defects in X65 grade pipelines using the ABAQUS finite element software. The effects of corrosion depth, length, and spacing on the pipeline failure pressure were systematically investigated. The main conclusions are summarized as follows:

(1) The axial spacing between double corrosion defects significantly influences the pipeline failure pressure, and a critical axial spacing exists. When the spacing is less than the critical value, the failure pressure decreases markedly; when the spacing exceeds the critical value, the interaction becomes negligible and the two defects can be treated as independent.

(2) The effect of circumferential spacing between double corrosion defects on failure pressure is relatively weak. For every 10° increase in circumferential spacing, the variation in failure pressure is merely 0.05–0.01 MPa. Furthermore, when the circumferential spacing exceeds approximately 10°, the interaction is essentially negligible and the double corrosion defects can be treated as two independent single defects.

(3) Based on the numerical simulation results, empirical expressions, relating to the failure pressure of axially and circumferentially aligned double corrosion defects to their spacing, were derived. The fitting correlation coefficients $R^{2}$ are 0.97805 and 0.99964, respectively, indicating good agreement between the fitted results and the simulation data.

## Figures and Tables

**Table 1:** Parameters of corrosion defects.

Pipeline Material	Relative Corrosion Depth M	Corrosion Length (mm)	Corrosion Width (°)	$\mathrm{Corrosion}\;\mathrm{Axial}\;\mathrm{Spacing}\;S_{l}$ (mm)
X65	0.2	200	20	10/20/40/60/80/100
	0.4	200	20	10/20/40/60/80/100
	0.6	200	20	10/20/40/60/80/100

**Table 2:** Failure pressure of single-point corroded pipeline (X65, corrosion length 200 mm, corrosion width 20°).

Pipeline Material	Relative Corrosion Depth M	Corrosion Width (°)	Failure Pressure (MPa)
X65	0.2	20	20.0
	0.4	20	18.0
	0.6	20	16.0

Note: The values in the table are calculated using the same finite element model as in Table 1 and are rounded to one decimal place.

**Table 3:** Parameters of corrosion defects.

Pipeline Material	Relative Corrosion Length N	Relative Corrosion Depth M	Corrosion Width (°)	$\mathrm{Axial}\;\mathrm{Corrosion}\;\mathrm{Spacing}\;S_{l}$ (mm)
X65	0.5	0.4	20	10/20/40/60/80/100
	1.0	0.4	20	10/20/40/60/80/100
	2.0	0.4	20	10/20/40/60/80/100
	3.0	0.4	20	10/20/40/60/80/100

**Table 4:** Parameters of corrosion defects.

Pipe Material	Relative Corrosion Depth M	Corrosion Length (mm)	Corrosion Width (°)	$\mathrm{Circumferential}\;\mathrm{Corrosion}\;\mathrm{Spacing}\;S_{w}$ (°)
X65	0.2	200	20	10/20/30/40/50/60
	0.4	200	20	10/20/30/40/50/60
	0.6	200	20	10/20/30/40/50/60

**Table 5:** Parameters of corrosion defects.

Pipeline Material	Relative Corrosion Length N	Relative Corrosion Depth M	Corrosion Width (°)	$\mathrm{Circumferential}\;\mathrm{Corrosion}\;\mathrm{Spacing}\;S_{w}$ (°)
X65	0.5	0.4	20	10/20/30/40/50/60
	1.0	0.4	20	10/20/30/40/50/60
	2.0	0.4	20	10/20/30/40/50/60
	3.0	0.4	20	10/20/30/40/50/60

**Table 6:** Fitting results of each parameter.

**Index**	a	b	c	d	e	$R^{2}$	SSE
Fitting Result	0.66975	1.85298	−0.63971	−0.33592	0.12546	0.97805	9.4366

**Table 7:** Fitting results of each parameter.

**Index**	a	b	c	d	e	$R^{2}$	SSE
Fitting Result	0.984	0.01558	−0.2054	0.36155	0.55876	0.99964	0.11072

**Table 8:** Comparison of parameters for double corrosion defect studies.

Pipe Type	Relative Corrosion Depth	Corrosion Width (°)	Failure Pressure (MPa)
Yang Yanhua et al. (X100)	0.35/0.44/0.52/0.61/0.70/0.79	approx. 2.6–6.1	Decreases by 1–2.5 MPa per 2 mm increase in depth *
Chen Fei (X80)	0.2/0.3/0.4/0.5/0.6/0.7/0.8	approx. 7.9	16.4–25.1 (varies with spacing and depth)
Present study (X65)	0.2/0.4/0.6	20	Double-point axial spacing 10 mm: 18.1/15.0/12.1

* Note: Yang Yanhua et al. did not provide a baseline single-point failure pressure under a unified working condition; the tabulated values represent the sensitivity trend with respect to corrosion depth.

## Data Availability

The original contributions presented in this study are included in the article. Further inquiries can be directed to the corresponding authors.
